# Disease burden and treatment patterns of paroxysmal nocturnal hemoglobinuria in Japan: a real-world survey

**DOI:** 10.1007/s12185-026-04170-w

**Published:** 2026-02-11

**Authors:** Masatoshi Sakurai, Yasutaka Ueda, Naoshi Obara, Michel Kroes, Takeo Dochi, Anggie Wiyani, Maria-Magdalena Balp, Olivier Somenzi, Yasmin Taylor, Tatsuya Kawaguchi, Jun-ichi Nishimura

**Affiliations:** 1 Division of Hematology, Department of Medicine, Keio University School of Medicine, 35 Shinanomachi, Shinjuku-ku, Tokyo, 160-8582 Japan; 2 Department of Hematology and Oncology, The University of Osaka Graduate School of Medicine, Suita, Japan; 3 Department of Medical Sciences, Laboratory Hematology, Faculty of Medicine, University of Tsukuba, Ibaraki, Japan; 4 Novartis Pharma AG, Basel, Switzerland; 5 Novartis Pharma Tokyo, Tokyo, Japan; 6 Novartis Pharmaceuticals UK Ltd, London, UK; 7 Adelphi Real World, Bollington, UK; 8 Kumamoto Health Science University, Kumamoto, Japan

**Keywords:** Paroxysmal hemoglobinuria, Complement inhibitors, Disease burden

## Abstract

This study aimed to determine the clinical profile and disease burden of patients with paroxysmal nocturnal hemoglobinuria (PNH) in Japan using real-world data from the Adelphi Real World PNH Disease Specific Programme (DSP)™, a cross-sectional survey conducted between January and December 2022. Data included demographics, treatment, and clinical values (hemoglobin [Hb] and lactate dehydrogenase [LDH]). Patient-reported outcome measures included the EQ-5D-5L and the FACIT-Fatigue. Seventeen physicians provided information on 45 patients, among whom 86.7% were receiving treatment with complement 5 inhibitors (C5i). Median (IQR) age was 65.0 (54.5, 73.0) years; 55.6% were male. Among C5i-treated patients (n = 39), 51.3% received ravulizumab and 48.7% eculizumab, for a median (IQR) duration of 1.6 (1.0, 2.7) years. Median (IQR) Hb level was 7.2 (7.0, 8.1) g/dL at diagnosis and 10.0 (8.8, 10.6) g/dL at survey; 91.4% had LDH levels exceeding 1.5 times the upper limit of normal at diagnosis, 11.4% at survey. Twelve patients returned a self-completed questionnaire. Patient-reported symptoms included tiredness (83.3%), shortness of breath (75.0%), and anemia (58.3%). Mean (SD) EQ-5D-5L and FACIT-Fatigue scores were 0.73 (0.16) and 32.3 (7.1). Even C5i-treated patients continued to experience substantial disease burden, highlighting the need for more effective treatments to improve quality of life.

## Introduction

Paroxysmal nocturnal hemoglobinuria (PNH) is a rare, acquired hematological disorder characterized by chronic intravascular hemolysis and thrombosis [1]. Globally, the prevalence of PNH has been estimated to be around 15.9 cases per million individuals [2]. Patients with PNH often experience a multitude of symptoms including anemia, fatigue, dyspnea, hemoglobinuria, and abdominal pain [3]. These symptoms can significantly impact patients’ health-related quality of life (HRQoL), overall well-being, as well as the ability to carry out daily activities and employment [4].

In Japan, the first treatments available for PNH were complement 5 inhibitors (C5i), including eculizumab in 2010 [5], followed by ravulizumab in 2019 [6]. Post-marketing follow-up studies of patients with PNH in Japan have demonstrated the sustained safety and effectiveness of both eculizumab and ravulizumab, which is consistent with clinical trial data [7–9]. Generally, treatment with C5i has improved the prognosis for PNH patients by reducing fatigue, hemolysis, the need for transfusions, the risk of thrombosis or renal failure, and has improved long-term survival [10].

However, persistent symptoms despite C5i treatment may result from several factors, including residual intravascular hemolysis and/or the emergence of C3-mediated extravascular hemolysis [11], leading to suboptimal responses in a significant proportion of patients [12]. A cross-sectional survey of patients with PNH in Japan has demonstrated that despite treatment with C5i, many patients are still experiencing a significant disease burden [13].

With the availability of newer treatments for PNH in Japan, and given the significant challenges and unmet clinical needs faced by patients, gaining a more comprehensive understanding of the overall disease burden is increasingly important. However, real-world studies examining the burden of PNH in Japan are scarce. Therefore, this study aimed to describe the clinical profile, disease burden, and management of patients with PNH in Japan using real-world data.

## Methods

### Study design

This study utilized Japanese data abstracted from the Adelphi Real World PNH Disease Specific Programme (DSP)™, a large, multinational, cross-sectional survey of physicians and their patients, conducted between January and December 2022. The DSP methodology has been previously described [14, 15], validated [16], and demonstrated to be representative and consistent over time [17].

### Physician-reported data

Physicians (hematologists, oncologists, and hematologist-oncologists) were eligible to participate in the DSP provided they were personally responsible for the treatment decisions and management of patients with PNH. Physicians were recruited from secondary hematology services (public or private hospitals, clinics, or offices) in Japan through publicly available lists.

Physicians were asked to complete a patient record form for their next ten consecutively consulting adult patients with PNH. Patient record forms contained detailed questions on patients’ sociodemographics; comorbidities; disease and treatment history, current management; clinical values including hemoglobin (Hb), lactate dehydrogenase (LDH) [categorized as above or below 1.5× the upper limit of normal (ULN), where the ULN was defined as 250 U/L], and absolute reticulocyte count, which were all collected at diagnosis and at time of survey; signs and symptoms; treatment goals, treatment satisfaction and preference for treatment administration; and healthcare resource utilization. For laboratory parameters, the last value closest to the time of survey was used as a proxy for “value at time of survey”.

Data for clinical values were reported on a matched basis, where values were available both at the time of diagnosis and at the time of survey. When assessing satisfaction, physicians were asked to select from one of the following five response options: completely dissatisfied, dissatisfied, neither satisfied nor dissatisfied, satisfied, or completely satisfied.

### Patient-reported data

Patients were then invited to voluntarily complete a patient self-complete questionnaire, reporting demographics, signs and symptoms, treatment goals and several patient-reported outcomes measures such as the EQ-5D-5L with EQ5D-Visual Analogue Scale (VAS), Medical Outcomes Study 36-Item Short Form Survey version 1 (SF-36), Functional Assessment of Chronic Illness Therapy-Fatigue (FACIT-Fatigue) and the Work Productivity and Activity Impairment (WPAI). Some signs and symptoms response options reflected conditions that the patient were already aware they had; for example, “anemia” represented the patients’ understanding of having been diagnosed with anemia, rather than a laboratory-defined measurement. Patients were eligible for inclusion if they were ≥18 years old and had a physician-confirmed diagnosis of PNH.

#### EQ-5D-5L and VAS

EQ-5D-5L measures generic HRQoL on the day of assessment across five domains: mobility, self-care, usual activities, pain/discomfort, and anxiety/depression [18, 19]. Utility scores, calculated with Japan-tariffs [20], range from 1 (full health) to 0 (a health state equivalent to death). VAS is a scale (range from 0 to 100) where respondents indicate their overall health status on the day of completion.

#### SF-36

The SF-36 assesses HRQoL across the following eight domains: physical functioning, role-physical, bodily pain, general health, vitality, social function, role-emotional, and mental health. The recall period is 4 weeks. Domain scores range from 0 to 100, with higher scores indicating better health. Two composite scores are created based on respective domain scores: the Physical Component Summary (PCS) score and the Mental Component Summary (MCS) score [21–23]. Both PCS and MCS scores are interpreted relative to the general population, with a mean of 50 and standard deviation of 10. Scores above 50 indicate better-than-average HRQoL, while scores below 50 indicate below-average health in the respective physical or mental domain.

#### FACIT-fatigue

The FACIT-Fatigue questionnaire, with a recall period of 7 days, evaluates the impact of disease and treatment on fatigue in patients with chronic illnesses such as PNH. Scores range from 0 to 52, with higher scores indicating lower levels of fatigue [24].

#### WPAI

The WPAI measures impairments of work and activities over a period of 7 days, and is interpreted through four impairment scores expressed as percentages: absenteeism, presenteeism, overall work impairment (composite score of absenteeism and presenteeism), and overall activity impairment [25]. Scores range from 0 to 100% with higher scores meaning higher impairment. Due to low sample size, only activity impairment is reported in this analysis.

### Data analysis

Analyses were descriptive; categorical variables are presented as frequency and percentage, and continuous variables presented as means and standard deviations (SD) and/or medians with interquartile ranges (IQR). Analyses were conducted in Stata Statistical Software version 17.0.5 (StataCorp. 2021. College Station, TX: StataCorp LLC).

### Ethical considerations

Patients provided informed consent to take part in the survey via a checkbox, and data were collected in such a way that patients and physicians could not be identified directly. Physician and patient data were pseudo-anonymized and aggregated to mitigate against tracing the individual.

Data collection was undertaken in line with European Pharmaceutical Marketing Research Association guidelines [26] and, as such, did not require ethics committee approval. The study materials were submitted to the Pearl Institutional Review Board (IRB) and received ethical exemption (IRB protocol number #21-ADRW-127). Each survey was performed in full accordance with relevant legislation at the time of data collection, including the US Health Insurance Portability and Accountability Act 1996 [27].

## Results

### Physician-reported data

#### Patient demographics and clinical characteristics

A total of 17 physicians provided information on 45 patients with PNH (overall cohort). Of these patients, 39 (86.7%) were receiving treatment with C5i at the time of survey (C5i cohort).

Overall (n = 45), patients had a median (IQR) age of 65.0 (54.5, 73.0) years, 55.6% were male, and mean (SD) BMI was 21.9 (2.7) kg/m^2^ (Table 1). At the time of survey, 35.6% of patients were in employment. The median (IQR) duration between a diagnosis of PNH to the time of survey completion was 3.3 (1.6, 6.4) years. The most common comorbidities at the time of survey included hypertension (35.6%) and aplastic anemia (22.2%), and most comorbidities were present prior to the onset of PNH symptoms, including aplastic anemia.

**Table 1:** Patient demographics and clinical characteristics

	Overall cohort n = 45	C5i cohort n = 39
Age (years), median (IQR)	65.0 (54.5, 73.0)	65.0 (55.0, 74.0)
Sex, n (%)		
Male	25 (55.6)	20 (51.3)
BMI (kg/m^2^), mean (SD)	21.9 (2.7)	22.2 (2.7)
Employment, n (%)		
Working full-time	14 (31.1)	12 (30.8)
Unemployed	12 (26.7)	8 (20.5)
Homemaker	9 (20.0)	9 (23.1)
Retired	6 (13.3)	6 (15.4)
Working part-time	2 (4.4)	2 (5.1)
On long-term sick leave	1 (2.2)	1 (2.6)
Student	1 (2.2)	1 (2.6)
Time since PNH diagnosis (years)		
Patients with data, n	44	39
Median (IQR)	3.3 (1.6, 6.4)	3.3 (1.7, 6.6)
Comorbidities at the time of survey^a^, n (%)		
Patients with data, n	45	39
Hypertension	16 (35.6)	14 (35.9)
Aplastic anemia	10 (22.2)	8 (20.5)
Gastroesophageal reflux disease	5 (11.1)	2 (5.1)
Congestive heart failure	5 (11.1)	4 (10.3)

C5i, C5 inhibitor; IQR, interquartile range; PNH, paroxysmal nocturnal hemoglobinuria; SD, standard deviation

^a^Comorbidities reported for ≥10% of total sample

#### Management of PNH

Of the C5i cohort (n = 39), 51.3% were receiving ravulizumab and 48.7% were receiving eculizumab at the time of survey (Table 2). Patients had been receiving their current prescription of C5i for a median (IQR) of 1.6 (1.0, 2.7) years. A third (33.3%) of patients were prescribed concomitant medication alongside C5i therapy. Physicians reported that the most common concomitant medications prescribed to patients in the C5i cohort were anticoagulants (15.4%), corticosteroids (15.4%), and dietary supplements (7.7%).

**Table 2:** Physician-reported management of PNH

	Overall cohort n=45	C5i cohort n=39
*Treatments received at the time of survey*^*a*^*, n (%)*		
Ravulizumab	20 (44.4)	20 (51.3)
Eculizumab	19 (42.2)	19 (48.7)
Corticosteroids	8 (17.8)	6 (15.4)
Anticoagulants	6 (13.3)	6 (15.4)
Hormonal therapy	6 (13.3)	1 (2.6)
Immunosuppressive therapy	3 (6.7)	2 (5.1)
Dietary supplements	3 (6.7)	3 (7.7)
Other	3 (6.7)	3 (5.1)
Analgesic medication	2 (4.4)	1 (2.6)

C5i, C5 inhibitor; SD, standard deviation

^a^Treatments are not mutually exclusive, which means patients may have received multiple treatment classes concurrently.

^b^Other treatments include eltrombopag and iron chelating agent

For the overall cohort of patients (n = 44), over a quarter (27.3%) received at least 1 blood transfusion in the 12 months prior to time of survey. Of those, patients had a mean (SD) 6.6 (6.0) blood transfusions. For patients who were diagnosed for ≥12 months (n=38), 23.7% received one or more transfusions, with a mean (SD) of 6.2 (5.9) in the past 12 months. Of patients in the C5i cohort (n = 38), 28.9% received one or more blood transfusions in the 12 months prior to survey, with a mean (SD) of 5.7 (5.5). For patients who had been prescribed C5i for ≥12 months (n = 30), 23.3% received one or more transfusions and the mean (SD) was 6.6 (6.6).

#### Clinical profile

**Fig. 1:**
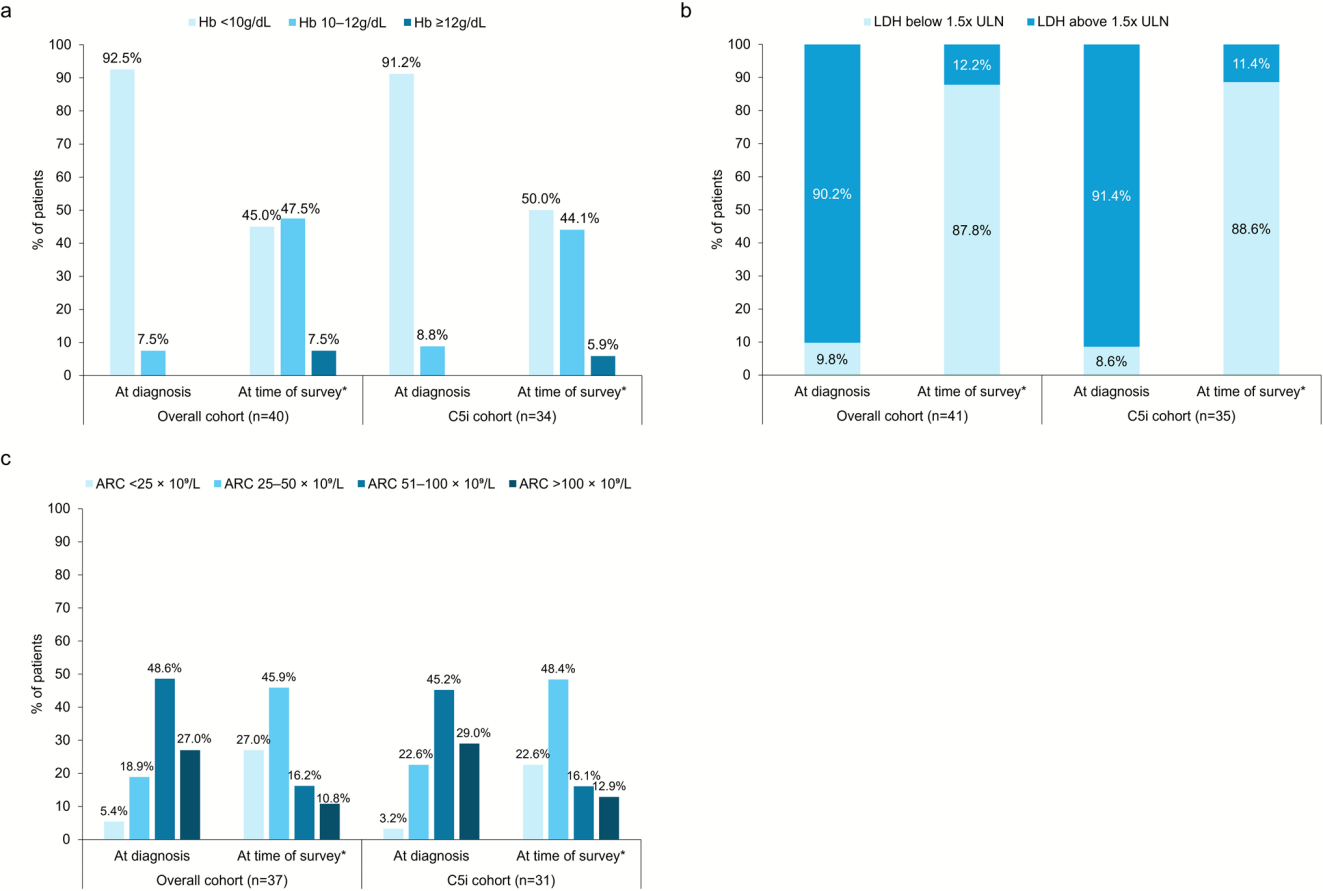
Clinical profile of the overall and the C5i cohorts. **a** Hemoglobin levels at diagnosis and at time of survey. **b** Lactate dehydrogenase levels (U/L) 1.5 × ULN at diagnosis and at time of survey. **c** Absolute reticulocyte count at diagnosis and at time of survey. *The last value closest to the time of survey was used as a proxy for value “at time of survey.”. ULN defined as 250 U/L. Abbreviations: ARC, absolute reticulocyte count; Hb, hemoglobin; LDH, lactate dehydrogenase; ULN, upper limit of normal

Of the overall cohort (n = 40), the median (IQR) Hb level was 7.2 (7.0, 8.1) g/dL at diagnosis and 10.0 (9.0, 10.6) g/dL at time of survey. For the C5i cohort (n = 34), median (IQR) Hb level was 7.2 (7.0, 8.1) g/dL at diagnosis, and 10.0 (8.8, 10.6) g/dL at time of survey. Only 7.5% of patients in the overall cohort and 5.9% of patients in the C5i cohort achieved Hb levels ≥12 g/dL at the time of the survey (Fig. 1). At diagnosis, LDH levels above 1.5× the upper limit of normal (ULN) were observed in 90.2% of the overall cohort (n = 41) and 91.4% of the C5i cohort (n = 35); at the time of survey, this was observed in 12.2 and 11.4% of patients, respectively. Absolute reticulocyte count (ARC) was >100 × 10^9^/L in 27.0% of patients in the overall cohort (n = 37) at diagnosis, and 10.8% at the time of survey; the proportion of patients with ARC >100 × 10^9^/L in the C5i cohort (n = 31) was 29.0% at diagnosis, and 12.9% at the time of survey.

**Fig. 2:**
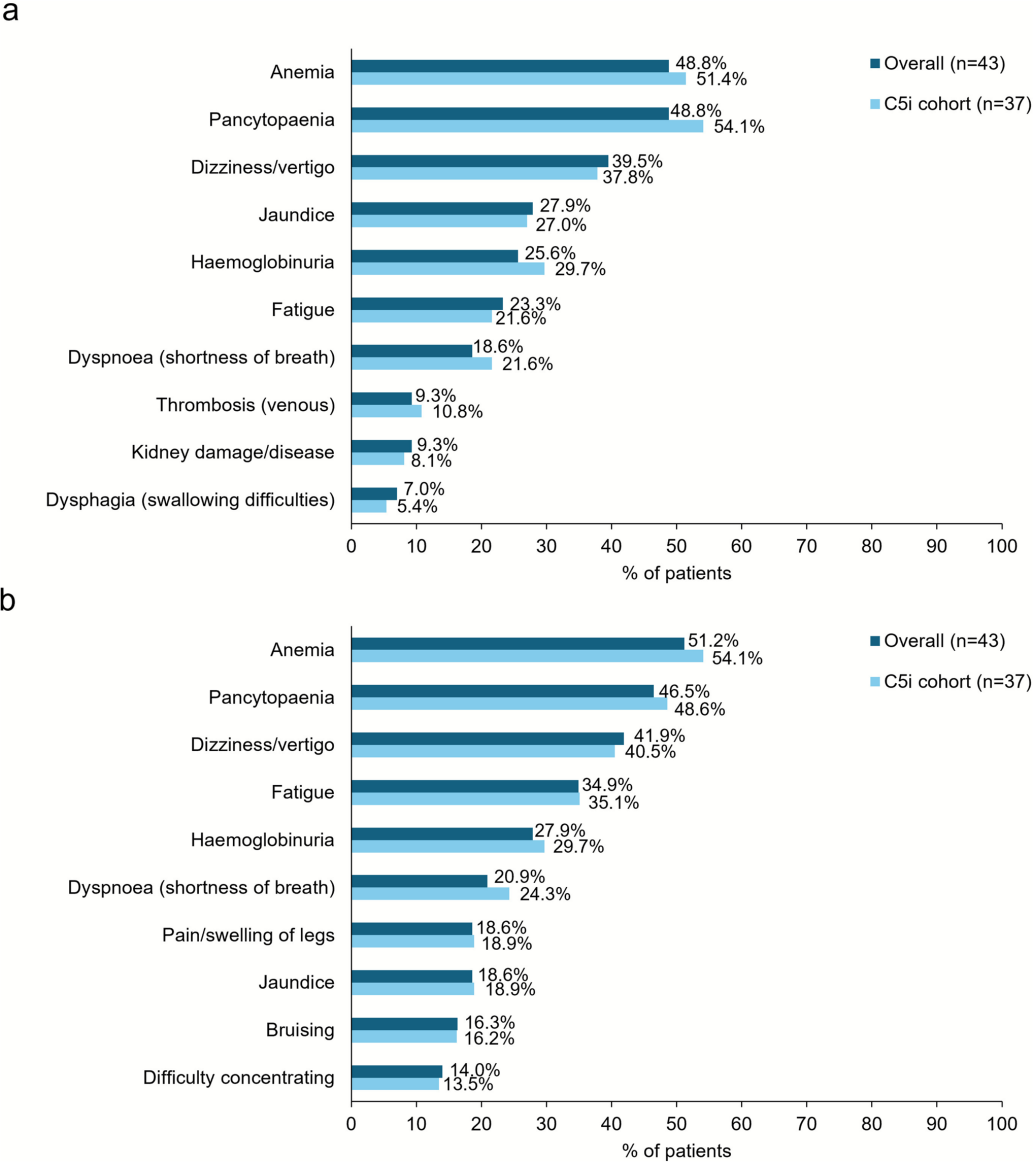
Physician-reported signs and symptoms of PNH. Top 10 physician-reported symptoms for the overall and C5i cohorts **a** at time of PNH diagnosis and **b** at the time of survey.

Overall, physicians reported that the most common signs and/or symptoms experienced by their patients at the time of diagnosis and at the time of survey, respectively, were anemia (48.8 and 51.2%), pancytopenia (48.8 and 46.5%), dizziness (39.5 and 41.9%), fatigue (23.3 and 34.9%), and hemoglobinuria (25.6 and 27.9%); results observed for the C5i cohort were comparable to the overall cohort (Fig. 2).

#### Physician goals and satisfaction with their patients PNH treatment

Of the overall cohort (n = 45), the most common primary goals of treatment, as reported by physicians for their patients, were increasing Hb levels (62.2%), reducing the need for blood transfusions (44.4%), reducing LDH levels (40.0%), prevention or reduction of anemia (37.8%), and prevention of thrombotic events (35.6%). When asked which treatment goal was the most important, physicians selected preventing thrombotic events (17.8%), increasing hemoglobin levels (17.8%), followed by preventing or reducing anemia (15.6%). Overall, 62.2% of physicians were satisfied or completely satisfied with their patients’ treatment regimen at the time of survey. Physicians were satisfied or completely satisfied with treatment for 85.0% of patients receiving ravulizumab (n = 20), and 36.8% of patients receiving eculizumab (n = 19).

**Fig. 3:**
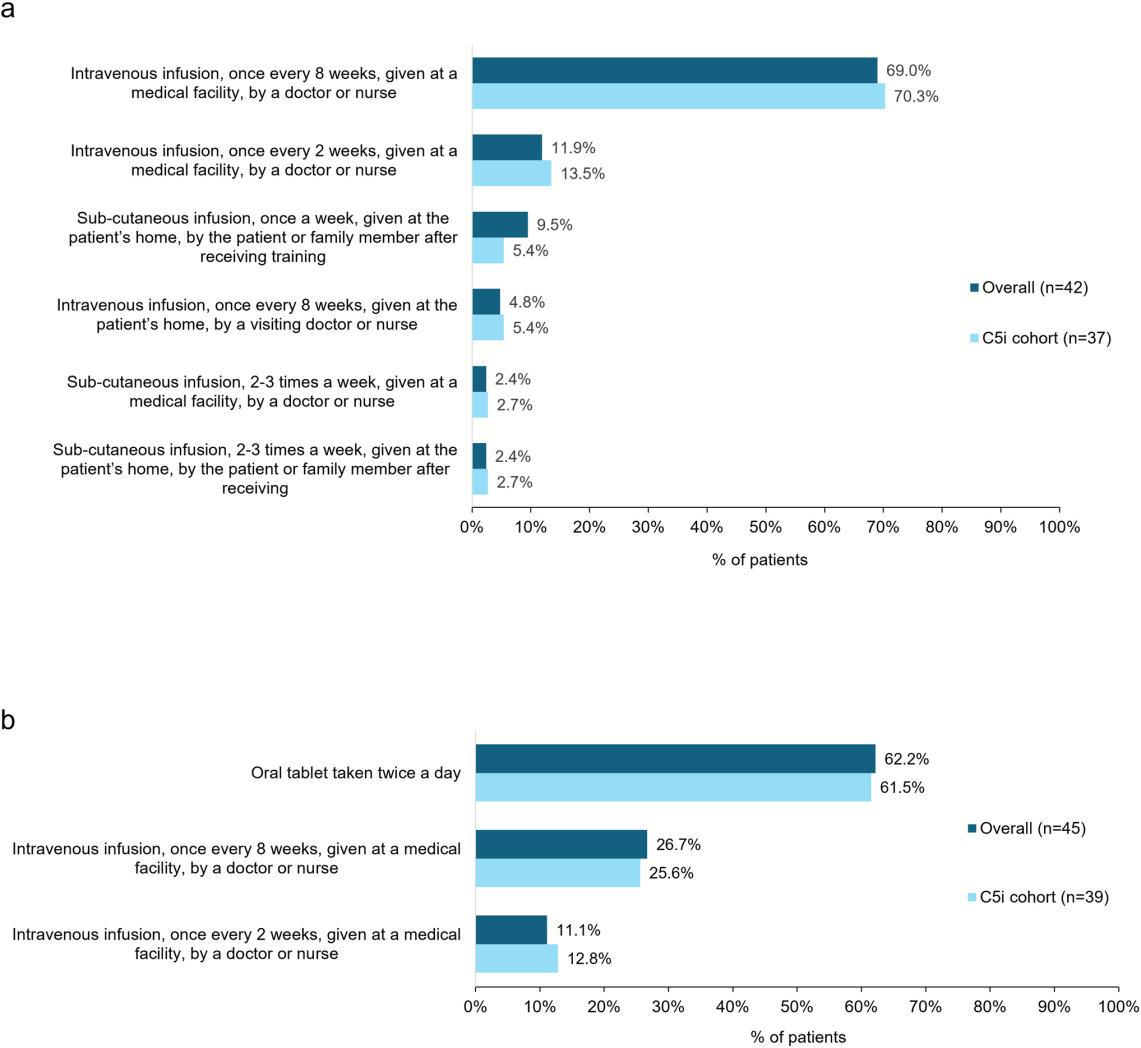
**a** Physician-reported preferred route of treatment administrations for available treatments at the time of survey and **b** Physician-reported preferred route of treatment administrations if alternative formulations became available

Physicians were asked about their preferred route of treatment administration for treatments available at the time of survey for their patients (n = 42). The majority (69.0%) of physicians reported a preference for intravenous infusion administered once every 8 weeks, followed by intravenous infusion administered once every 2 weeks (11.9%), each administered in a medical facility by a doctor or nurse (Fig. 3a). Physicians were further asked to select their preferred route of administration should alternative formulations become available; 62.2% expressed a preference for an oral formulation taken twice daily, followed by intravenous infusion every 8 weeks (26.7%), or every 2 weeks (11.1%; Fig. 3b).

#### Healthcare resource utilization

Overall, the mean (SD) number of healthcare professionals (HCPs) involved in PNH management was 1.2 (0.6). The three most common HCPs involved in PNH management were hematologists (73.3%), hematologist–oncologists (26.7%), and emergency room (ER) physicians (6.7%). The mean (SD) number of visits to any HCP in the 12 months prior to survey was 10.4 (8.4) visits.

Overall (n = 43), 25.6% of patients had been hospitalized at least once in the 12 months prior to the time of survey (Table 3). For the most recent hospitalization, 54.5% of patients were admitted through an ER, and patients spent a mean (SD) number of 7.5 (4.5) nights in hospital. The most common reasons for the most recent hospitalization were infection (63.6%), severe fatigue (18.2%), and thrombotic event (9.1%). For patients within the C5i cohort, 24.3% were hospitalized due to their PNH at least once within the 12 months prior to time of survey. For the most recent hospitalization, 55.6% of patients were admitted through the ER and spent a mean (SD) of 8.7 (4.1) nights in hospital.

**Table 3:** Physician-reported healthcare resource utilization

	Overall cohort n=45	Diagnosed for ≥12 months n=39	C5i cohort n=39	Prescribed C5i treatment for ≥12 months n=31
*Number of PNH-related hospitalizations in the 12 months prior to survey*				
Patients with data, n	43	37	37	29
Mean (SD)	0.3 (0.7)	0.3 (0.7)	0.2 (0.4)	0.2 (0.4)
≥ 1 hospitalizations, n (%)	11 (25.6)	8 (21.6)	9 (24.3)	5 (17.2)
*Reason for most recent hospitalization*				
Patients with data, n	11	8	9	5
Infection, n (%)	7 (63.6)	6 (75.0)	6 (66.7)	3 (60.0)
Severe fatigue, n (%)	2 (18.2)	0 (0.0)	1 (11.1)	0 (0.0)
Thrombotic event, n (%)	1 (9.1)	1 (12.5)	1 (11.1)	1 (20.0)
Other, n (%)	1 (9.1)	1 (12.5)	1 (11.1)	1 (20.0)
*Admitted via ER for most recent hospitalization*				
Patients with data, n	11	8	9	5
Yes, n (%)	6 (54.5)	4 (50.0)	5 (55.6)	2 (40.0)
No, n (%)	5 (45.5)	4 (50.0)	4 (44.4)	3 (60.0)
*Number of nights spent in most recent hospitalization*				
Patients with data, n	11	8	9	5
Mean (SD)	7.5 (4.5)	8.6 (4.7)	8.7 (4.1)	9.6 (4.0)

C5i, C5 inhibitor; ER, emergency room; PNH, paroxysmal nocturnal hemoglobinuria; SD, standard deviation

Of patients diagnosed for ≥12 months, 21.6% had been hospitalized in the 12 months prior to the time of survey. For the most recent hospitalization, 50.0% were admitted through the ER and spent a mean (SD) of 8.6 (4.7) nights (Table 3). For patients prescribed a C5i for ≥12 months, 17.2% had at least one hospitalization in the 12 months prior to time of survey. For the most recent hospitalization, 40.0% of patients were admitted via the ER, and spent a mean (SD) of 9.6 (4.0) nights in hospital.

### Patient-reported data

#### Patient-reported treatment goals, signs and symptoms, and impact of PNH

Overall, a total of 12 patients returned a self-complete questionnaire. Of these patients, 58.3% (n = 7) were prescribed a C5i at the time of survey, all of which were receiving eculizumab.

**Fig. 4:**
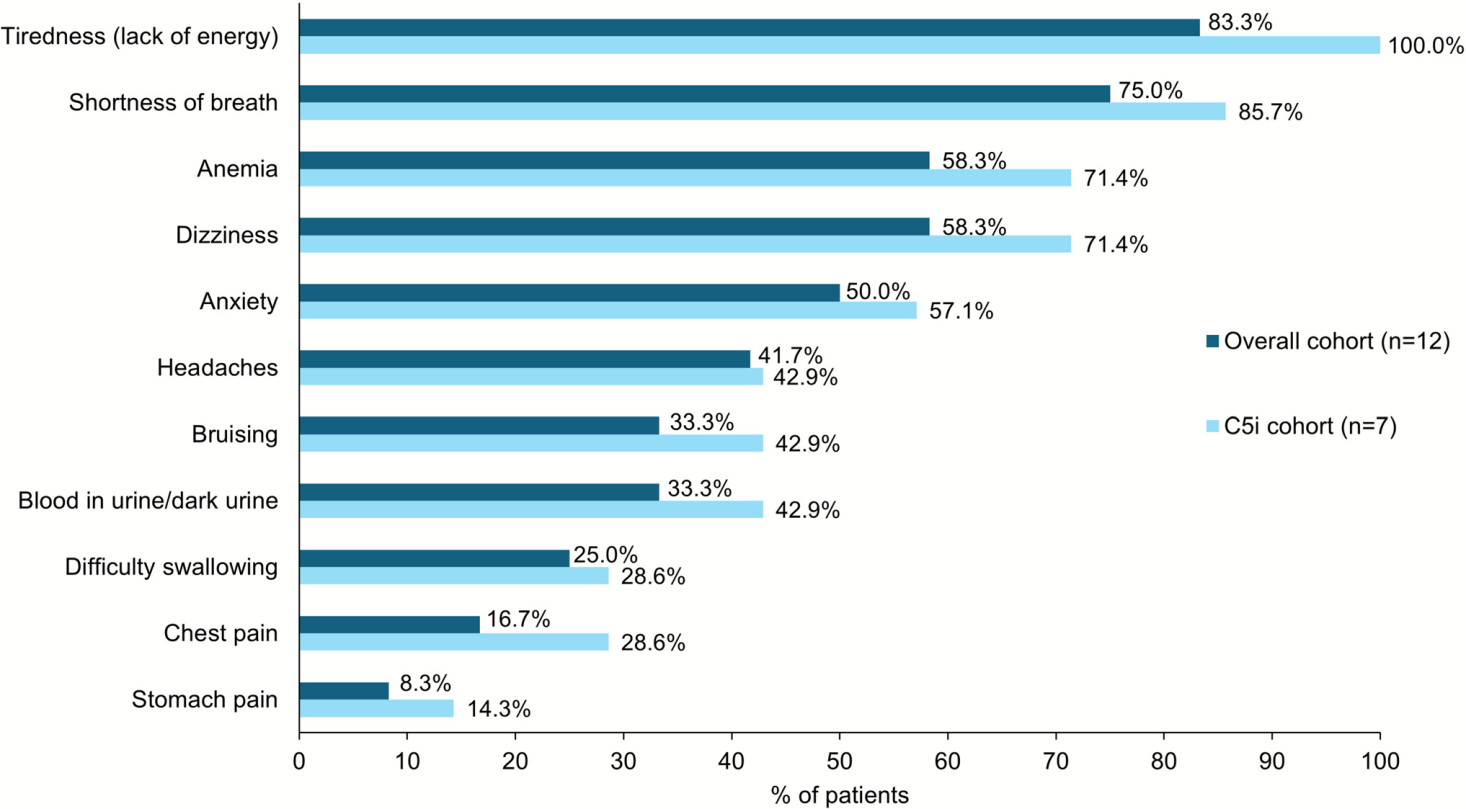
Patient-reported symptoms at the time of survey

Overall, the most common signs and symptoms reported by patients at the time of survey were tiredness (83.3%), shortness of breath (75.0%), anemia (58.3%) and dizziness (58.3%; Fig. 4). The mean (SD) EQ-5D VAS score was 67.9 (19.0) and EQ-5D-5L utility score was 0.73 (0.16). The health status on the SF-36 showed a mean (SD) PCS score of 43.9 (9.6) and MCS score of 43.3 (8.4). The mean (SD) FACIT-Fatigue score was 32.3 (7.1). The mean (SD) overall activity impairment on WPAI was 38.3% (30.1%).

All patients were asked about their preference for route of treatment administration; an oral PNH-treatment was preferred by 83.3% of patients and intravenous infusion was preferred by 16.7%. Patients reported that their most important treatment goals were reducing tiredness (63.6%) and allowing for a ‘normal life’ (18.2%).

## Discussion

Our real-world survey examined the clinical profile, disease management, and disease burden of patients with PNH in Japan. We found that despite treatment with C5i therapy, only 6% of patients had normalized Hb [12], with levels ≥12 g/dL at the time of survey. These findings highlight an unmet need of patients prescribed C5i in regard to normalizing Hb and LDH levels, and align with a previous cross-sectional study conducted in Japan, which found 59% of patients reported Hb levels <10.5 g/dL despite receiving C5i for more than a year [13]. Persistent anemia in patients receiving C5i has been widely attributed to mechanisms beyond terminal complement blockade. C3-mediated extravascular hemolysis may occur despite adequate inhibition of intravascular hemolysis, as C5i do not prevent upstream opsonization of erythrocytes [28]. Additionally, underlying bone marrow failure can limit erythroid recovery even when hemolysis is controlled [28]. These processes are well recognized contributors to persistent anemia in treated PNH patients and may explain the proportion of patients in our study who did not achieve normal Hb levels. Over a quarter of patients with PNH in our study required at least one blood transfusion, and 24% underwent at least one hospitalization in the 12 months prior to survey, despite treatment with C5i. This was corroborated by an analysis of retrospective claims databases in the US which also found that hospitalization was common among patients with PNH, with 40% having an inpatient admission, and 23% requiring blood transfusions [29].

Although we found that most physicians reported satisfaction with their patients PNH treatment, including C5i, they also reported that patients continued to experience symptoms such as anemia, pancytopenia, dizziness, fatigue, and dyspnea. The continued prevalence of these symptoms among patients currently receiving C5i treatment, as demonstrated in our findings, suggests that existing treatments may not adequately address the clinical manifestations of the disease. In our study, the most common signs and symptoms reported by patients were tiredness, shortness of breath, and anemia, with patients also identifying the reduction of tiredness as their top goal of treatment. Persistent symptoms, such as tiredness and shortness of breath, among patients receiving C5i are likely multifactorial. Incomplete hemoglobin recovery, comorbidities, and the psychosocial burden of chronic disease can further contribute to symptom persistence. Our findings are consistent with previous studies, which have shown that fatigue and dyspnea are among the most common symptoms both at the time of diagnosis and the time of survey [13]. Physician-reported treatment satisfaction, considered alongside patient-reported symptoms, may suggest that while therapies align with clinical expectations, they may not fully alleviate the patients’ symptom burden. Taken together, these findings underscore the need for new treatments that provide more comprehensive symptom control, to ultimately improve overall quality of life for patients.

Our findings highlight the disease burden and impact on HRQoL among patients in Japan receiving C5i. The mean FACIT-Fatigue score of 32.3 suggests a considerable level of fatigue within this population, which remains a key concern despite treatment, and is consistent with prior research [30]. Similarly, the mean EQ-5D-5L utility score of 0.73 indicated impaired HRQoL among patients. A previous study reported a mean EQ-5D-5L score of 0.86 in a similar patient population, and the general population norms in Japan have been reported as 0.936 for men and 0.928 for women, which suggests that patients with PNH are experiencing notable disease-related burden [13, 31]. The mean EQ-5D VAS score, indicating overall health status on the day of survey, of 67.9, further reflects the perceived burden experienced by patients. Collectively, these findings highlight the need for comprehensive care strategies that address both clinical and quality of life aspects of disease in patients with PNH.

Several approaches have the potential to support improved quality of life in patients with PNH. Optimizing disease management through established treatment pathways and incorporating emerging therapies, such as proximal complement inhibition or oral complement inhibitors, may alleviate symptom burden for some patients. In clinical practice, improved patient–physician communication can help physicians to better understand individual priorities, treatment goals, and daily challenges, enabling more tailored decisions around disease management. The use of symptom-tracking tools may further facilitate early recognition of evolving symptoms and enhance shared decision-making [32]. Together, these strategies may contribute to more patient-centered care and improved quality of life.

### Limitations

As with all survey methodologies, the DSP has several limitations. Minimal inclusion criteria governed the selection of participating physicians; therefore, participation was influenced by their willingness to complete the survey. Physicians were asked to provide data for a consecutive series of patients to avoid selection bias; however, in the context of a rare disease like PNH, the feasibility of capturing ten consecutive patients is not always possible. This may have introduced selection bias, as physicians with a higher PNH caseload were more likely to be eligible and participate. As a result, the findings may not be representative of the broader PNH population, particularly those receiving C5i, and may overrepresent patients with more severe disease or those who consult with their physician more frequently. The DSP did not capture information on the timing of the most recent C5i treatment; therefore, patients may have been at different points within their dosing interval when laboratory values were reported, potentially contributing to variability in Hb levels. Furthermore, as the DSP reflects the consulting population, patients with persistent symptoms or suboptimal responses to therapy may be more likely to attend regular consultations with their physician and as such, may be overrepresented. Recall bias may also have affected the responses of both patients and physicians, which is a common limitation of surveys. However, the data for these analyses were collected at the time of each patient’s consultation and physicians had access to patient medical records, which reduces the likelihood of recall bias. In addition, patient-reported data were available from only 12 respondents, of whom 7 were prescribed complement therapy—all treated with eculizumab, which further limits the generalizability of these findings, particularly in terms of patient experience.

### Conclusion

This study described the real-world impact of PNH on patients in Japan who, despite receiving C5i treatment, continued to require blood transfusions and hospitalization in many cases. The findings suggest that patients may still experience a substantial burden of disease, with persistent anemia and fatigue commonly reported. Taken together, the results underscore a continued unmet need for treatments that offer more comprehensive control of hemolysis, normalize LDH and Hb levels, and restore HRQoL, thereby potentially reducing the long-term burden of PNH on patients.

## Data Availability

All data, i.e. methodology, materials, data and data analysis, which support the findings of this survey are the intellectual property of Adelphi Real World. All requests for access should be addressed directly to Yasmin Taylor at yasmin.taylor@adelphigroup.com.

## References

[1] Brodsky RA. Paroxysmal nocturnal hemoglobinuria. Blood. 2014;124(18):2804–11. 25237200 10.1182/blood-2014-02-522128 PMC4215311

[2] Sahin F, Ozkan MC, Mete NG, Yilmaz M, Oruc N, Gurgun A, et al. Multidisciplinary clinical management of paroxysmal nocturnal hemoglobinuria. Am J Blood Res. 2015;5(1):1–9. 26171279 PMC4497492

[3] Schrezenmeier H, Röth A, Araten DJ, Kanakura Y, Larratt L, Shammo JM, et al. Baseline clinical characteristics and disease burden in patients with paroxysmal nocturnal hemoglobinuria (PNH): updated analysis from the International PNH Registry. Ann Hematol. 2020;99(7):1505–14. 32390114 10.1007/s00277-020-04052-z PMC7316848

[4] Escalante CP, Chisolm S, Song J, Richardson M, Salkeld E, Aoki E, et al. Fatigue, symptom burden, and health-related quality of life in patients with myelodysplastic syndrome, aplastic anemia, and paroxysmal nocturnal hemoglobinuria. Cancer Med. 2019;8(2):543–53. 30632713 10.1002/cam4.1953 PMC6382725

[5] Ministry of Health, Labour and Welfare. Pharmaceuticals and Medical Devices Agency. Soliris (eculizumab). 2010. (Accessed 15/10/2025) Available from: https://www.pmda.go.jp/files/000153009.pdf..

[6] Ministry of Health, Labour and Welfare. Pharmaceuticals and Medical Devices Agency. Ultomiris (ravulizumab). 2019. (Accessed 15/10/2025) Available from: https://www.pmda.go.jp/drugs/2022/P20220825002/870056000_30100AMX00022_B100_1.pdf..

[7] Ikezoe T, Noji H, Ueda Y, Kanda Y, Okamoto S, Usuki K, et al. Long-term follow-up of patients with paroxysmal nocturnal hemoglobinuria treated with eculizumab: post-marketing surveillance in Japan. Int J Hematol. 2022;115(4):470–80. 35146630 10.1007/s12185-022-03287-y

[8] Nishimura J-i, Kawaguchi T, Ito S, Murai H, Shimono A, Matsuda T, et al. Real-world safety profile of eculizumab in patients with paroxysmal nocturnal hemoglobinuria, atypical hemolytic uremic syndrome, or generalized myasthenia gravis: an integrated analysis of post-marketing surveillance in Japan. Int J Hematol. 2023;118(4):419–31. 37515657 10.1007/s12185-023-03630-x

[9] Usuki K, Ikezoe T, Ishiyama K, Kanda Y, Gotoh A, Hayashi H, et al. Interim analysis of post-marketing surveillance of ravulizumab for paroxysmal nocturnal hemoglobinuria in Japan. Int J Hematol. 2023;118(3):311–22. 37477863 10.1007/s12185-023-03625-8

[10] Hillmen P, Young NS, Schubert J, Brodsky RA, Socié G, Muus P, et al. The complement inhibitor eculizumab in paroxysmal nocturnal hemoglobinuria. N Engl J Med. 2006;355(12):1233–43. 16990386 10.1056/NEJMoa061648

[11] Risitano AM, Marotta S, Ricci P, Marano L, Frieri C, Cacace F, et al. Anti-complement treatment for paroxysmal nocturnal hemoglobinuria: time for proximal complement inhibition? A position paper from the SAAWP of the EBMT. Front Immunol. 2019;10:1157. 31258525 10.3389/fimmu.2019.01157 PMC6587878

[12] Debureaux P-E, Kulasekararaj AG, Cacace F, Silva BGP, Calado RT, Barone F, et al. Categorizing hematological response to eculizumab in paroxysmal nocturnal hemoglobinuria: a multicenter real-life study. Bone Marrow Transplant. 2021;56(10):2600–2. 34226670 10.1038/s41409-021-01372-0

[13] Obara N, Usuki K, Hayashi T, Fujii M, Ikezoe T. Burden of illness in Japanese patients with paroxysmal nocturnal hemoglobinuria receiving C5 inhibitors. Int J Hematol. 2024;119(3):255–64. 38243016 10.1007/s12185-023-03698-5 PMC10920411

[14] Anderson P, Benford M, Harris N, Karavali M, Piercy J. Real-world physician and patient behaviour across countries: disease-specific programmes - a means to understand. Curr Med Res Opin. 2008;24(11):3063–72. 18826746 10.1185/03007990802457040

[15] Anderson P, Higgins V, Courcy J, Doslikova K, Davis VA, Karavali M, et al. Real-world evidence generation from patients, their caregivers and physicians supporting clinical, regulatory and guideline decisions: an update on disease specific programmes. Curr Med Res Opin. 2023;39(12):1707–15. 37933204 10.1080/03007995.2023.2279679

[16] Babineaux SM, Curtis B, Holbrook T, Milligan G, Piercy J. Evidence for validity of a national physician and patient-reported, cross-sectional survey in China and UK: the disease specific programme. BMJ Open. 2016;6(8):e010352. 27531722 10.1136/bmjopen-2015-010352 PMC5013497

[17] Higgins V, Piercy J, Roughley A, Milligan G, Leith A, Siddall J, et al. Trends in medication use in patients with type 2 diabetes mellitus: a long-term view of real-world treatment between 2000 and 2015. Diabetes Metab Syndr Obes. 2016;9:371–80. 27843332 10.2147/DMSO.S120101 PMC5098530

[18] EuroQolGroup. EuroQol--a new facility for the measurement of health-related quality of life. Health Policy. 1990;16(3):199–208. 10109801 10.1016/0168-8510(90)90421-9

[19] Herdman M, Gudex C, Lloyd A, Janssen M, Kind P, Parkin D, et al. Development and preliminary testing of the new five-level version of EQ-5D (EQ-5D-5L). Qual Life Res. 2011;20(10):1727–36. 21479777 10.1007/s11136-011-9903-x PMC3220807

[20] Roudijk B, Ludwig K, Devlin N. EQ-5D-5L Value Set Summaries. In: Devlin N, Roudijk B, Ludwig K, editors. Value Sets for EQ-5D-5L: a compendium, comparative review & user guide. Cham (CH): Springer. 2022, pp. 55–212. 36810025

[21] Ware JE, Sherbourne CD. The MOS 36-item short-form health survey (SF-36). I. Conceptual framework and item selection. Med Care. 1992;30(6):473–83. 1593914

[22] McHorney CA, Ware JE, Raczek AE. The MOS 36-Item Short-Form Health Survey (SF-36): II. Psychometric and clinical tests of validity in measuring physical and mental health constructs. Med Care. 1993;31(3):247–63. 8450681 10.1097/00005650-199303000-00006

[23] McHorney CA, Ware JE, Lu JF, Sherbourne CD. The MOS 36-item Short-Form Health Survey (SF-36): III. Tests of data quality, scaling assumptions, and reliability across diverse patient groups. Med Care. 1994;32(1):40–66. 8277801 10.1097/00005650-199401000-00004

[24] Yellen SB, Cella DF, Webster K, Blendowski C, Kaplan E. Measuring fatigue and other anemia-related symptoms with the functional assessment of cancer therapy (FACT) measurement system. J Pain Symptom Manage. 1997;13(2):63–74. 9095563 10.1016/s0885-3924(96)00274-6

[25] Reilly MC, Zbrozek AS, Dukes EM. The validity and reproducibility of a work productivity and activity impairment instrument. Pharmacoeconomics. 1993;4(5):353–65. 10146874 10.2165/00019053-199304050-00006

[26] EphMRA. European Pharmaceutical Market Research Association (EphMRA) Code of Conduct 20192019. Available from: http://www.ephmra.org/Code-of-Conduct-Support..

[27] HIPPA. Summary of the HIPAA Privacy Rule. United States Department of Health and Human Services (US HHS); 2003

[28] Risitano AM, de Peffault Latour R. How we('ll) treat paroxysmal nocturnal haemoglobinuria: diving into the future. Br J Haematol. 2022;196(2):288–303. 34355382 10.1111/bjh.17753 PMC9291300

[29] Clayton D, Shafrin J, Yen G, Lee S, Geevarghese L, Shi Y, et al. Treatment patterns and healthcare resource utilization of patients with paroxysmal nocturnal hemoglobinuria: a retrospective claims data analysis. Clin Appl Thromb Hemost. 2024;30:10760296231213073. 38173351 10.1177/10760296231213073 PMC10768575

[30] Cella D, Johansson P, Ueda Y, Tomazos I, Gustovic P, Wang A, et al. Clinically important change for the FACIT-Fatigue scale in paroxysmal nocturnal hemoglobinuria: a derivation from international PNH registry patient data. J Patient Rep Outcomes. 2023;7(1):63. 37405515 10.1186/s41687-023-00609-4 PMC10322798

[31] Shiroiwa T, Fukuda T, Ikeda S, Igarashi A, Noto S, Saito S, et al. Japanese population norms for preference-based measures: EQ-5D-3L, EQ-5D-5L, and SF-6D. Qual Life Res. 2016;25(3):707–19. 26303761 10.1007/s11136-015-1108-2 PMC4759213

[32] Griffiths EA, Min JS, Lee WN, Yu JC, Patel Y, Myren KJ, et al. Patient-reported outcomes and daily activity assessed with a digital wearable device in patients with paroxysmal nocturnal hemoglobinuria treated with ravulizumab: reveal, a prospective, observational study. Health Qual Life Outcomes. 2024;22(1):62. 39123253 10.1186/s12955-024-02279-2 PMC11313122

